# Deconvolution of Acoustic Emission and Other Causal Time Series

**DOI:** 10.6028/jres.096.019

**Published:** 1991

**Authors:** John A. Simmons

**Affiliations:** National Institute of Standards and Technology, Gaithersburg, MD 20899

**Keywords:** acoustic emission, causal time series, cross-cut algorithm, deconvolution, FFT deconvolution, Gaussian deconvolution, root projection, signal processing, singular value decomposition, time series, transducer calibration

## Abstract

A new technique, root projection (RP), is given for quantitative deconvolution of causal time series in the presence of moderate amounts of noise. Deconvolution is treated as a well-conditioned but underdetermined problem and *a priori* information is employed to obtain comparable noise reduction to that achieved by singular value decomposition (SVD) techniques while providing more accurate frequency information about the inverse. Two detailed examples arc given. The first gives noise analysis for alternate methods for deconvolution with a Gaussian kernel. The second example presents a model acoustic emission transducer calibration problem with typical noisy and incomplete output data. This example is treated by the use of a robust cross-cutting algorithm combining both the RP and SVD methods.

## 1. Introduction

Because of the almost ubiquitous presence of Green’s functions in linear physical theories, de-convolution (or inverse filtering) is the most commonly encountered special inverse problem associated with many characterization problems in NDE, both electromagnetic and elastic. A difficulty with inverse problems is their susceptibility to ill-conditioning; and, as is well-known, deconvolution problems can range in difficulty from those simply solvable by forward substitution to those for which no known method holds any hope of solution.

Some of the most powerful techniques that can be applied to deconvolution are those, such as regularization and singular valued decomposition (SVD), arising from general linear inversion methods. However, there are other methods available which take advantage of the special properties of the convolution process. We shall review briefly some of these methods, compare them with an SVD method especially adapted for deconvolution and present a new approach to deconvolution resting on the concept of root projection which, when combined with SVD, offers the possibility of approaching currently intractable deconvolution problems.

Two examples—both ill-conditioned—will be presented, and signal-to-noise concepts will be introduced to sharpen the SVD and root projection deconvolution (RPD) methods. These will then be compared with more standard deconvolution methods. The first example involves convolution with a Gaussian kernel in which the entire output is given, but corrupted by varying amounts of noise. In the second example the convolution kernel is derived from the actual response of a “standard” acoustic emission transducer in a calibration experiment. As is typical in many such experiments, the entire output is not available. So one is faced with deconvolving a one-sided kernel from an incomplete one-sided time series output. To treat this problem, the SVD and RPD methods are combined into a more robust cross-cutting algorithm which provides both superior time and frequency information about the inverse.

## 2. Deconvolution Methods

Full convolution of two finite-length, real-valued, one-sided time series may be expressed as

a:N*b:M=(a*b):(M+N), (1)

where ***a*** is a causal time series with elements *a*_0_,*…*,*a_N_*, ***b*** *is a* causal time series with elements *b*_0_,…,*b_M_*, and where a*b is a causal time series with elements^1^

$${\left(a*b\right)}_{n}=\sum_{k}a_{k}b_{n-k},\;n=0,\cdots ,M+N.$$ (2)

The fact that ***a:b*** or ***b:M*** are truncated at finite length makes no difference to the first min(*M,N*) terms in the output.

In the deconvolution problem one is given the series ***a:N*** and some part of the right hand series in eq (1), usually *M* terms, which is possibly corrupted by noise. For most applications one assumes that *M=N*, if need be by zero padding the shorter truncated series, and one writes the deconvolution problem in the form

a:N*b:N=υ:N (3)

where ***a:N*** and ***υ:N*** are assumed known and ***β:N*** is to be reconstructed as closely as possible. The series ***a:N*** is called the kernel series. In *Y* transform terminology eq (1) may be paraphrased as

$$a\left(y\right)\beta \left(y\right)=\upsilon \left(y\right)+y^{N+1}u\left(y\right),$$ (4)

where ***u*:**(*N* − 1) is usually an unknown time series.

In the notation used in eq (1) with *N < M*, if ***a:N*** is thought of as an *N*th order difference operator, then eq (1) becomes a high order difference equation with the first *K* terms associated with the boundary conditions at the causal boundary and the last *N−K −* 1 terms associated with the boundary conditions at the transient boundary. Treating eq (3), then, has the interpretation of solving a one point boundary value problem, while eq (4) may be thought of as solving the two point boundary value problem where the influence of the transient boundary conditions on the solution may be studied and these conditions possibly restricted using *a priori* information.

In *Y* transform terms the inversion of eq (4) merely amounts to finding the coefficients of the Taylor’s series of (*υ*(*y*) + *u* (*y*)*y^N^*^+1^)/*a*(*y*) about the origin, and the first *N +*1 of these coefficients are the same as those of *υ*(*y*)/*a*(*y*) so long as *a*_0_≠0. From this point of view the expansion of υ(*y*)/*a*(*y*) about the origin is only valid within the radius of convergence of the Taylor’s expansion. Since *υ*(*y*) is a truncated series and has noise, its zeroes will not include those of *a*(*y*), so that this expansion is generally only valid to the innermost zero of *a* (*y*).

Let the modulus of the smallest zero of *a*(*y*) be *r*_0_. If we use a circle of radius *r < r*_0_, then we can write the Cauchy formula for the Taylor’s expansion coefficients as

$$\beta_{n}=\frac{1}{2\pi i}\oint_{\left|y\right|=r}\frac{\upsilon \left(y\right)}{a\left(y\right)}dy.$$ (5)

Approximating this integral by numerical evaluation on *r* times the *M*th roots of unity gives:

$$\beta_{n}=\frac{r^{-n}}{M}\sum_{k=0}^{M-1}\frac{\upsilon }{a}\left(re^{\;\frac{2\pi ki}{M}}\right)\;e^{\;-\frac{2\pi ki}{M}}.$$ (6)

This formula is exact when 0 ≼ *n* ≼ *N < M* if ***υ:N*** = (a*β):*N*. Otherwise, all coefficients $\beta_{n}^{\prime }$, *n′ ≡m* (mod *M*) of the Laurent expansion of *υ*(*y*)/*a*(*y*) will be aliased onto *β_n_.*

If one defines the “radiused” time series $\hat{ar}:N$, where ${\left(\hat{ar}\right)}_{n}=a_{n}r^{n},\;n=0,\cdots ,N$, then eq (6) is merely a DFT inversion formula on the quotient of the DFT’s of the radiused time series $\hat{vr}:N$ by $\hat{ar}:N$ where the *n* th term of the inverted series is multiplied by the expansion factor of *r*^−^*^n^*. We refer to this technique implemented with the FFT algorithm as radiused FFT inversion. The factor *r*^−^*^n^* in eq (6) expands noise exponentially rendering this algorithm generally useless in its raw form. The algorithm can, however, be employed in conjunction with root projection. That application will be discussed in detail shortly.

If one attempts to employ eq (6) for *r < r*_0_ to reduce the noise buildup, one obtains the expansion of the wrong Laurent series form of *υ/a*, a form yielding a non-causal time series whose terms depend on the residues of *υ*(*y*) at the roots of *a*(*y*) inside *r.* In moving *r* from the inside to the outside of any one of these roots one changes the contribution due to that root from a causal time series with a factor of the residue times *r*^−^*^n^* into an anticausal time series with terms of the type residue times *r^n^*^−1^. In a DFT context these terms also wrap around through negative times to produce time aliasing, especially for large positive times. The rapidity of convergence of the partial fraction series associated with a given root *y_α_* depends on that ratio *y_α_/r* or *r/y_α_* which is less than one.^2^

In matrix notation the deconvolution problem is almost exclusively formulated in the parallel form of eq (3):

$$\left[\begin{array}{lllllll} a_{0} & 0 & . & . & . & 0 & 0\\ a_{1} & a_{0} & . & & & . & .\\ . & a_{1} & . & . & & . & .\\ . & . & & . & . & . & .\\ . & . & & & . & 0 & .\\ a_{N-1} & . & . & . & . & a_{0} & 0\\ a_{N} & a_{N-1} & . & . & . & a_{1} & a_{0} \end{array}\right]\;\left[\begin{array}{l} \beta_{1}\\ \beta_{2}\\ .\\ .\\ .\\ \beta_{N-1}\\ \beta_{N} \end{array}\right]=\left[\begin{array}{l} \upsilon_{0}\\ \upsilon_{1}\\ .\\ .\\ .\\ \upsilon_{N-1}\\ \upsilon_{N} \end{array}\right]$$ (7)

while eq (4) takes the form:

$$\left[\begin{array}{lllllll} a_{0} & 0 & . & . & . & 0 & 0\\ a_{1} & a_{0} & . & & & . & .\\ . & a_{1} & . & . & & . & .\\ . & . & & . & . & . & .\\ . & . & & & . & 0 & .\\ a_{N-1} & . & . & . & . & a_{0} & 0\\ a_{N} & a_{N-1} & . & . & . & a_{1} & a_{0}\\ 0 & a_{N} & . & . & . & . & a_{1}\\ 0 & 0 & . & & & . & .\\ . & . & . & . & & . & .\\ . & . & & . & . & a_{N-1} & .\\ . & . & & & . & a_{N} & a_{N-1}\\ 0 & 0 & . & . & . & 0 & a_{N} \end{array}\right]\;\left[\begin{array}{l} \beta_{1}\\ \beta_{2}\\ .\\ .\\ .\\ \beta_{N-1}\\ \beta_{N} \end{array}\right]=\left[\begin{array}{l} \upsilon_{0}\\ \upsilon_{1}\\ .\\ .\\ .\\ \upsilon_{N-1}\\ \upsilon_{N}\\ u_{1}\\ u_{2}\\ .\\ .\\ .\\ u_{N} \end{array}\right]$$ (8)

Equation (7), while completely determined, is generally ill-conditioned. Equation (8), on the other hand, while underdetermined, is generally well-conditioned and offers new algorithms for dealing with the deconvolution problem.

The simplest method for solving eq (7) is by forward substitution, which is equivalent to formal division of the *Y* transforms *υ*(*y*)/*a*(*y*) starting at the constant term. This is generally the most unstable of inversion methods. The matrix equivalent of zero-padded and radiused FFT inversion is obtained by considering inverse-radiused circulant matrices.

From a real-valued time series *c:ℕ* we build the (*ℕ +*1) × (*ℕ +*1) inverse-radiused circulant matrix *C_r_* defined by:

$${\left(C_{r}\right)}_{kl}=r^{l-k}c_{\left(\mathrm{mod}\left(k-l,\mathbb{N}+1\right)\right)},k,l=0,\cdots ,\mathbb{N}.$$ (9)

It is then a simple matter to show that the vectors

$$\hat{r^{-1}e^{\;\frac{-2\pi mi}{N+1}}}:\mathbb{N}=\left[\begin{array}{c} 1\\ r^{-1}e^{\;\frac{-2\pi mi}{\mathbb{N}+1}}\\ r^{-2}e^{\;\frac{-4\pi mi}{\mathbb{N}+1}}\\ .\\ .\\ .\\ r^{-N}e^{\;\frac{-2\mathbb{N}\pi mi}{\mathbb{N}+1}} \end{array}\right]$$ (10)

are eigenvectors for *C_r_*. That is:

$$\begin{array}{l} \left[C_{r}\right]\hat{r^{-1}e^{\;\frac{-2\pi mi}{N+1}}}:\mathbb{N}=\left[{\left(\hat{e^{\;\frac{2\pi mi}{N+1}}:}\mathbb{N}\right)}^{T}c:N\right]\hat{r^{-1}e^{\;\frac{-2\pi mi}{N+1}}:}\mathbb{N}\\ \;={\bar{c}}_{m}\left(\hat{r^{-1}e^{\;\frac{-2\pi mi}{N+1}}}:\mathbb{N}\right), \end{array}$$ (11)

where $\tilde{c}:\mathbb{N}$ is the *ℕ +*1 point DFT of *c:ℕ.* In addition, the radiused DFT provides a decomposition of the discrete δ function:

$${\left(re^{\frac{2\pi mi}{N+1}}:\mathbb{N}\right)}^{T}r^{-1}e^{\frac{-2\pi mi}{N+1}}:\mathbb{N}=\left(\mathbb{N}+1\right)\delta_{mn}.$$ (12)

Then, the inversion of the equation:

C,β:ℕ=υ:ℕ (13)

is easily carried out by eigenvector decomposition:

$$\beta :\mathbb{N}=\frac{1}{\mathbb{N}+1}\sum_{m=0}^{\mathbb{N}}\frac{\left[{\left(r\hat{e^{\;\frac{2\pi mi}{N+1}}}:\mathbb{N}\right)}^{T}\upsilon :\mathbb{N}\right]}{{\tilde{c}}_{m}}\left(\hat{r^{-1}e^{\;\frac{-2\pi mi}{N+1}}}:\mathbb{N}\right).$$ (14)

To relate *c:ℕ* to *a:ℕ*, we set $c_{k}=a_{k}r^{k}$, *k* = 0,…,*N, c_k_* = 0, k *=N +* 1,…,*ℕ*, which is the radiused form of the zero-padded kernel series. Then the inverse-radiused matrix *C*, has the form shown in eq (15) of a

$$\left[\begin{array}{cccccccccccccccc} a_{0} & 0 & 0 & . & . & . & . & . & 0 & 0 & a_{N}r^{\left(\mathbb{N}+1\right)} & . & . & . & a_{2}r^{\left(\mathbb{N}+1\right)} & a_{1}r^{\left(\mathbb{N}+1\right)}\\ a_{1} & a_{0} & 0 & 0 & . & . & . & . & . & 0 & 0 & . & & & . & a_{2}r^{\left(\mathbb{N}+1\right)}\\ . & a_{1} & . & . & . & & & & & . & 0 & . & . & & . & .\\ . & . & . & . & . & . & & & & . & . & . & . & . & . & .\\ . & . & & . & . & . & . & & & . & . & & . & . & a_{N}r^{\left(\mathbb{N}+1\right)} & .\\ a_{N-1} & . & & & . & . & . & . & & . & . & & & . & 0 & a_{N}r^{\left(\mathbb{N}+1\right)}\\ a_{N} & a_{N-1} & . & . & . & a_{1} & a_{0} & 0 & 0 & . & . & & & & 0 & 0\\ 0 & a_{N} & . & . & . & . & a_{1} & a_{0} & 0 & 0 & . & & & & . & 0\\ . & 0 & . & . & & & & . & . & 0 & 0 & & & & . & .\\ . & . & . & . & . & & & & . & a_{0} & 0 & . & & & . & .\\ . & . & & . & . & . & & & & a_{1} & a_{0} & . & . & & . & .\\ . & . & & & . & . & . & & & . & a_{1} & . & . & . & . & .\\ . & . & & & & . & . & . & & . & . & . & . & . & 0 & .\\ . & . & & & & & . & . & . & . & . & & . & . & 0 & 0\\ 0 & 0 & . & . & . & . & . & 0 & a_{N} & a_{N-1} & . & . & . & . & a_{0} & 0\\ 0 & 0 & . & . & . & . & . & 0 & 0 & a_{N} & a_{N-1} & . & . & . & a_{1} & a_{0} \end{array}\right]$$ (15)

zero-padded convolution matrix with an extra exponentially damped upper right hand Toeplitz corner. Thus radiused FFT inversion solves the “damped” circulant rather than the convolution matrix problem. As seen from eqs (12) and (14), the answer obtained from this method will be correct for the convolution problem if *r*^(^*^N^*^+1)^ times the last *N* terms of the answer are effectively zero. That happy circumstance is usually frustrated by the exponential buildup in the eigenvectors of eq (10) together with the presence of noise components in ***υ:N***, as discussed previously in the *Y* transform context. However, referring to that discussion, one can conclude that if *r* < 1 and if there are no zeroes of *a*(*y*) with modulus less than *r*, then with sufficient zero padding FFT inversion will approach convolution inversion.

Least squares techniques are the standard way to deal with noise problems. Frequently they are formulated in terms of minimizing the norm ‖***υ:N*−A *β:N***‖. However, since ***υ:N*** contains signal-plus-noise, applying this condition too stringently forces induced domain noise into *β:N.* What is required is a technique to separate the range signal from the range noise either by applying *a priori* information about the signal in the range or *a priori* information about the signal in the domain. In a simple sense the example given in reference [2] giving rise to figure 4 accomplished this in the range using root projection when the complete information was available in the domain. We shall extend that concept below.

Another way to deal with this difficulty is through regularization, in which the norm is modified by adding another norm—usually a scalar times a domain norm—onto the above range norm to adjust the range residuals to be of the order of magnitude expected from the noise. The question is: Which norm should one add? The techniques for choosing an appropriate norm and carrying out this process efficiently lie somewhat outside the framework of this work, so that we shall not provide detailed comparisons with regularization methods here.

For eq (7) the least-squares method is usually implemented by multiplying the equation by *A^◊^* = (*A^T^*)^*^ to give the equation

$$A^{⋄}A\;\beta :N=A^{⋄}\upsilon :N$$ (16)

where *A^◊^A* is a non-negative Hermitian matrix. The multiplication accomplishes two results: *i*) It forces the output vector into the range of *A*, thereby eliminating some of the components of the noise, and *ii*) It modifies the left hand matrix to a Hermitian form allowing eigenvector decomposition methods and algorithms to be employed. However, this approach suffers from two drawbacks: *i*) lf *A* has a simple structure—e.g., *A* is a convolution operator — efficient algorithms which take advantage of this structure may be lost, and *ii*) The eigenvalues of *A^◊^A* are effectively the squares of those of *A* (in a meaning to be described directly), thus greatly increasing the ill-conditioned nature of the problem.

A closely related approach to the above is the singular value decomposition technique (SVD). In the SVD method one need not multiply the right hand side of eq (7) by *A*^◊^. Rather, one represents *A* in the form

$$A=U^{⋄}DV$$ (17)

where *U* and *V* are unitary matrices and *D* is a diagonal matrix whose diagonal elements are called the singular values of *A.* The SVD method, while usually slower to compute than the method arising from eq (15) has the advantage of finding diagonal elements which are the square roots of those stemming from *A^◊^A.* This permits greater dynamic range for treating very ill-conditioned problems such as frequently occur when solving eq (7). For the deconvolution problem the SVD algorithm has been implemented using an efficient form of another algorithm due to Lanczos (in addition to the SVD decomposition) to permit a small dimensional approximation to the full SVD approach [4]. However, this Lanczos/SVD approach, while more efficient may not permit accurate inversion especially when *N* is much greater than the dimension of the Lanczos subspace.

We turn now to least squares algorithms associated with eq (8). Calling *A* 2 the (2*N* + 1 × *N* + 1) matrix in that equation, one can say that *A*2 describes an embedding of the generally *N* +1 dimensional range of *A* in 2*N +* 1 dimensional space. In terms of the Euclidean inner products for both ${ℭ}^{2N+1}$ and ${ℭ}^{N+1}$ given by **(*u*,*υ***) = ***u****^T^*υ*, the adjoint of *A*2, *A*2^†^ *A*2^†^ = *A*2*^◊^ = A*2*^T^*, since.*A*2 is real [5]. Given a vector ***c:2N*** we can use *A*2^†^ to construct a least squares projection to map ***c:2N*** into the range *A*2, Once in the range, we shall see that inversion is well-conditioned and simple.

The necessary and sufficient condition that ***c:2N*** lie in the range of *A*2 is that ***c:2N*** lie in the orthogonal complement of the null space of *A*2^†^ [5]. That is:

$$\text{If}\;A\;2^{†}\;b:2N=0,\;\text{then}\;{\left(b:2N\right)}^{T}\bar{c:2N}=0.$$ (18)

To find a basis for the null space of *A*2^†^, we consider the geometric sequence vector $\hat{y}$ where *y* is any complex number. These vectors are eigenvectors of *A*2^†^ satisfying

$$A2^{†}\hat{y_{\alpha }}:2N={\left(\hat{y_{\alpha }}:2N\right)}^{T}A2=a\left(y\right)\hat{y_{\alpha }}:2N.$$ (19)

Thus, if *a* (*y*) has distinct roots, we can identify the *N* dimensional null space of *A*2^†^ as spanned by

$$\hat{y_{\alpha },}\;\text{where}\;a\left(y_{\alpha }\right)=0,\;\alpha =1,\cdots ,N.$$ (20)

The condition 17 then becomes the relation that *y_α_* are among the roots of *c*(*y*), or in algebraic terms *a*(*y*) divides *c*(*y*). From this point of view the existence of CBR implies a set of “continuity” equations that must be satisfied by time series in the range of *a*(*y*) under polynomial multiplication.

The root projection discussed in reference [2] provides us with the required mapping into the range of *A*2 [2]. Also, since there must exist a polynomial *b*(*y*) of degree *N* such that *a*(*y*)*b*(*y*) = *c*(*y*), we can find this polynomial simply by radiused FFT division with at least *2N +* 1 points using any numerically reasonable radius near one.

Thus we can formulate the root projection deconvolution (RPD) algorithm as an alternative least squares approach to deconvolution:

- Embedding ***υ:N*** in 2*N +* 1 dimensional space
  This is usually done by replacing an *a priori* range estimate with more accurate *a posteriori* range data where available. It can be done from the domain by convolving an *a priori* domain estimate with the kernel to give the *a priori* range estimate. Since the root projection of an *a priori* range estimate in this case is equal to itself, an equivalent method here is to subtract the *a priori* range estimate from the *a posteriori* range data, where available, and fill out the rest of the range with zeroes to produce a reduced problem with a zero *a priori* estimate. The *a priori* domain estimate can then be added back on to the inversion estimate from the reduced problem to give an upgraded inversion estimate. This latter approach can also be directly applied to introduce *a priori* information into the Lanczos/SVD method.
- Applying root projection
  Here time weighted inversion is employed using weightings on the *a priori* and *a posteriori* parts of the range data reflecting the relative uncertainties of the *a priori* versus *a posteriori* parts of the data. A very high weighting factor applied to the *a priori* part of the data will cause the root projection to return a result with very small residuals in the *a posteriori* part of the range data. This means that the inverse fits both the range signal and the superimposed noise more closely as one increases the ratio of weights of *a priori* to *a posteriori* points in the time weight file.
- Inverting using FFT division
  The entire FFT division operation is carried out on an *M* ≽ 2*N* + 1 dimensional space. The basis for this space is the set of DFT vectors built from the (*M* − 1)st roots of unity. However, only *N* of the *M* FFT components of any domain vector are independent, since there are *M −* (*N* + 1) conditions requiring all components after *N* + 1 to be zero. In the range there are still only *M −* (*N +* 1) FFT components independent. This time there are *N* conditions requiring orthogonality to the geometric root vectors of the kernel and *M−*(*N +* 1) conditions requiring all image space components (2*N +* 1) to be zero. Thus, the basic range projection consists of projecting through the set of geometric root vectors supplemented by any simple basis of the *M −* (*N +* 1) dimensional space of vectors whose first 2*N* +1 components are zero using the time (or frequency) weighted norm. The basic domain projection is through the *M−*(*N +* 1) dimensional space whose first *N* +1 components are zero. An independent orthogonal base for the range space is never built in root projection.

Few practical deconvolution problems can end at this point. Because of the presence of range noise, any inversion process will induce a domain noise which frequently obscures some or all of the features of the inverse. The critical problem when carrying out deconvolution consists of separating those aspects of the inverse which are “unequivocally” determined by the inversion process from those which are not. From the SVD or RPD point of view this means finding the signal-to-noise ratios in a series of orthogonal channels, accepting those aspects of the signal in the channels where the ratio is high and trying to supply *a priori* information in those where it is not. The SVD method provides an excellent means for optimal signal-to-noise separation. RPD, which works with frequency information, often provides an excellent representation for testing and including *a priori* information. One can add or remove various hypothetical features from an *a priori* estimate and test the consequence of these changes. Since with adequate weighting root projection always forces the range residuals back to within the noise limits, the result obtained will be a possible solution. However, essential solution features will be added and inessential ones taken away will not return.

Both RPD and SVD offer the possibility for determining the relative probability of a particular deconvolution estimate. However, we shall not discuss statistical algorithms in any systematic way here. Rather, we shall present two numerical examples principally to compare the accuracy and flexibility of the SVD, RPD, and radiused FFT division algorithms as inversion techniques and to present a cross-cut method for deconvolution combining Lanczos/SVD inversion (and filtering) with RPD and frequency space filtering.

In the first case discussed the noise we add is produced by rounding off the range data to a certain degree of accuracy, thus simulating an A/D conversion process. This noise while crudely uniform in frequency amplitude is not independent of the output, since one expects a negative correlation with the output derivative. In the second case the noise added was produced by a random number generator. Error will be measured by using a relative standard deviation. The ordinary standard deviation of one file from another will be divided by the generalized geometric mean of the non-zero terms of the reference file to give an order of magnitude correction to the error without being overly sensitive to “spikiness” in the reference file. Errors will only be compared for the same reference file.

## 3. Examples

### 3.1 Example I

**Figure 1:**
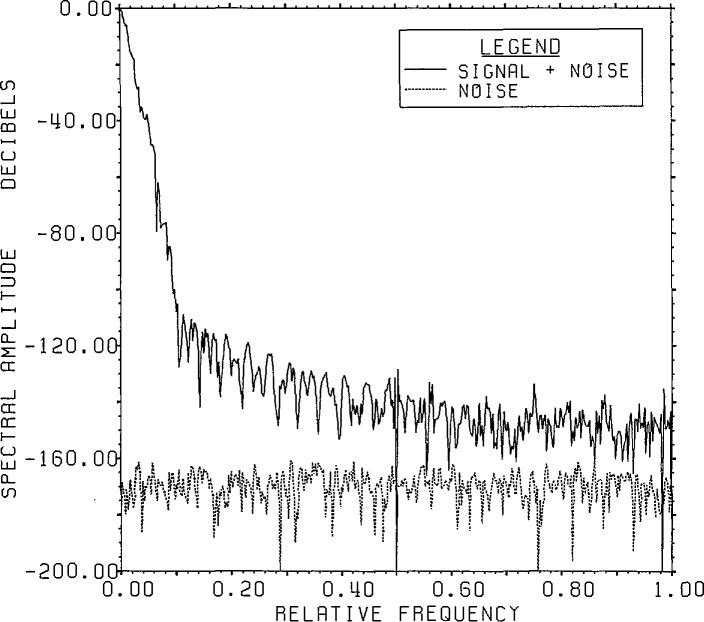
800 point FFT on the circle of radius 0.997 of the range signal-plus-noise and noise for the convolution of the time series in figures 2.4a[l] and 1[2] with 24 bit roundoff noise added (Example Ia).

We return to the example arising from the convolution of the 101 point Gaussian of figure 1 in reference [2] with the first 700 points of the experimental waveform of figure 2.4a in reference [1]. This example has the form of eq (1) with full range information; only noise has been added. This example illustrates one way of looking at *M +N* dimensional deconvolution in terms of root filtering. The 8 bit noise added on in that example was an “arbitrary” element of *M + N +* 1 dimensional space.

The projection condition placing the signal in the range of the kernel reduced the “power” of the noise by one eighth in this case. Another 700 degrees of freedom in the noise are connected to the roots of the *Y* transform of the answer, *β*(*y*). Although usually impractical, each piece of *a priori* information describing a root of *β*(*y*) removes one degree of freedom from the noise. When the roots of both the answer and the kernel are known, only the magnitude of the answer is undetermined. This is reflected in the fact that the only degree of freedom left for the noise is associated with the one-dimensional space generated by the convolution of the kernel and the answer, any element of which is orthogonal to the geometric series root vectors from both the kernel and the answer.

Two levels of range noise will be imposed for this example. We first consider the case where only 24 bit noise is added to the output, thus reducing the output to “single precision” accuracy.

Even with 24 bit accuracy the use of the forward substitution (or “real time deconvolution”) method for this problem produces errors in excess of 400% by term 100. The errors increase exponentially after that point.

Because most of the roots of the Gaussian lie on the unit circle, we carry out our analysis on a circle inside the unit circle. We choose a radius of 0.997 as reasonable for dealing with a series of length 700 and a zero-padded FFT of length 8192. Only two roots of the *Y* transform of the Gaussian lie inside this radius and the value of the *Y* transform of the noise at these roots is quite small so that in the case of 24 bit accuracy even radiused FFT inversion offers a good estimate of the answer if adequate zero padding is provided.

Figure 1 shows the range noise spectrum versus the spectrum of the range signal-plus-noise after root projection on the circle of radius 0.997.

Since the projected output lies in the range of the Gaussian under convolution, FFT division on a space of dimension 800 or greater is now accurate and each of the FFT components can be considered as lying in an orthogonal channel.^3^

Using the principle that throwing out a channel throws out both the signal and the noise in that channel, we have a simple criterion that we should reject all channels where the signal/noise ratio is less than one or, crudely, where the (signal-plus-noise)/noise ratio is less than $\sqrt{2}$. In this case that means essentially every channel is acceptable so that no filtering of the inverse should be necessary.

**Figure 2:**
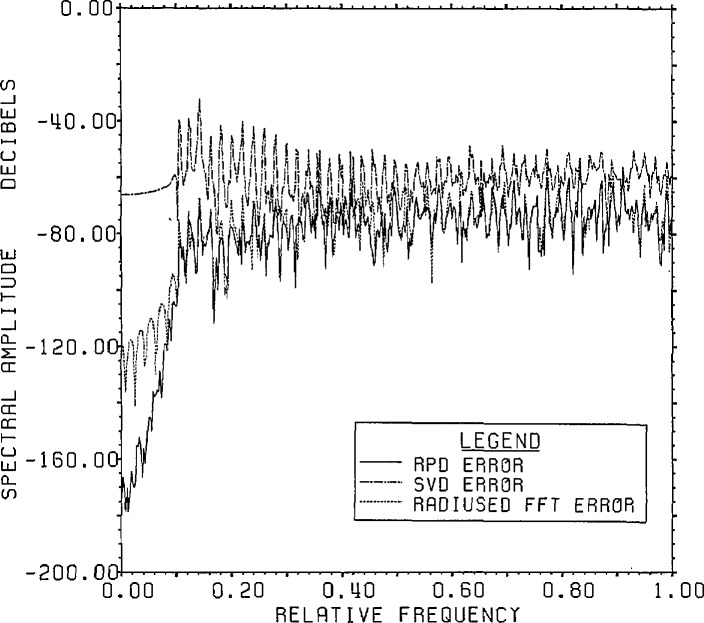
Comparison of spectra of errors for 3 deconvolution methods for Example Ia. An 800 point FFT was used on the circle of radius 0.997.

Figure 2 shows a comparison of the errors of the estimated inverse versus correct answer for RPD, radiused FFT inversion, and SVD inversion using a subspace of dimension 100. As can be seen, the RPD error is the smallest, merging with the FFT results for higher frequencies. The spectrum of the RPD error is, of course, exactly the quotient of the spectra of the range noise with that of the Gaussian kernel (padded to 800 points which was the FFT dimension used for RPD). The significantly larger error associated with the best Lanczos/SVD estimate arises from the use of only a 100 dimensional Lanczos subspace. The algorithm chosen uses the signal-plus-noise range vector as one of elements of the Lanczos subspace, but i)The range basis obtained may not be adequate to expand the noise or ii) The range basis may not separate the signal and the noise well, iii) The domain basis may not be adequate to expand the answer accurately, and iv) The matrix used to represent the convolution matrix has to be truncated at the last row perturbing it from its correct form on the whole space.

**Figure 3:**
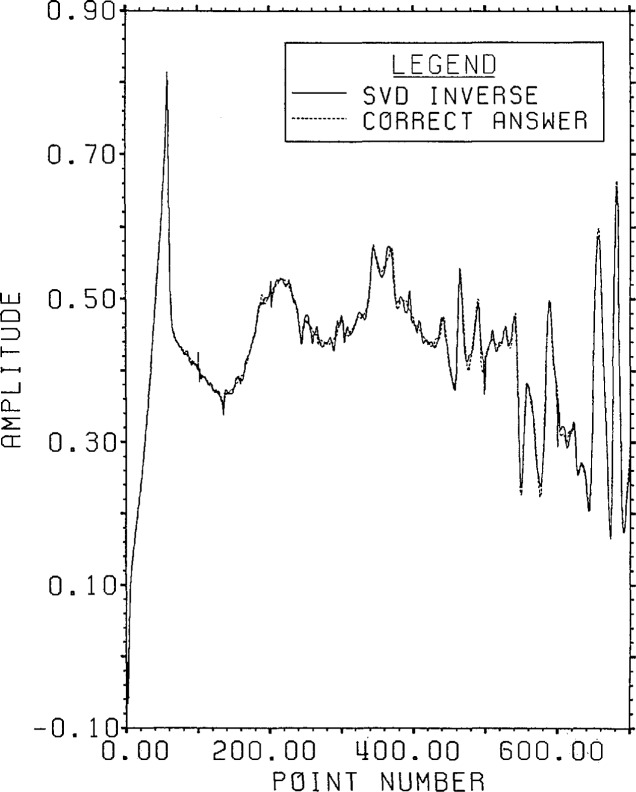
Example Ia: Overplot of the SVD inversion estimate and the correct answer.

In this case the error appears to stem equally from the last two of these causes. The relative standard deviation (r.s.d.) from the answer caused by inadequate domain basis is 1.6% while the r.s.d. for the best SVD estimate is 2.2%. Tne r.s.d. in expanding the noise is negligible. The two errors stand in the ratio about $\sqrt{2}$ which would be expected from equal independent contributions. By comparison the r.s.d. of the error of the RPD estimate is 0.16% while that of the FFT estimate is 0.25%. The relatively large error in the Lanczos/SVD estimate for this example remains no matter how small the roundoff error; only when the inversion error becomes small relative to the induced domain noise does the Lanczos/SVD method become effective. The SVD inversion estimate is shown in figure 3; the FFT and RPD estimates are visually identical to the correct answer.

**Figure 4:**
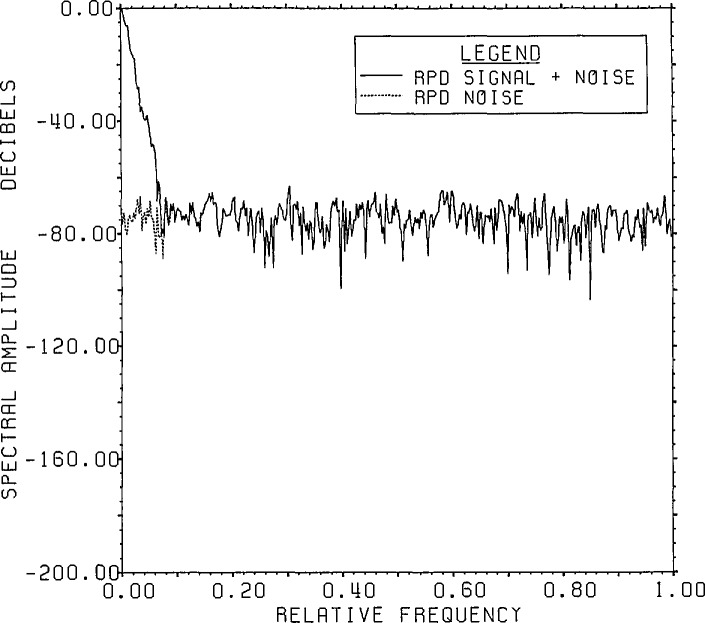
800 point FFT on the circle of radius 0.997 of the range signal-plus-noise and noise after RPD for the convolution of the time series in figures 2.4a [1] and 1 [2] with 8 bit roundoff noise added (Example Ib).

The ill-conditioned nature of the deconvolution process becomes apparent when the output has more noise. A comparison of range signal-plus-noise to noise after RPD is given in figure 4 for the case where the output has been truncated to 8 bit accuracy. The same figure made before root projection would appear visually to be substantially the same. The noise level is lowered about 0.6 dB by RPD. These spectra were also carried out on the circle of radius 0.997. Because the time curves were not renormalized after radiusing, the average noise level reads slightly below the −64 dB expected level for unradiused noise. As can be seen the (signal-plus-noise/noise) ratio is clearly greater than $\sqrt{2}$ until about 0.065 of the frequency band, dropping to less than one beyond 0.1.

**Figure 5:**
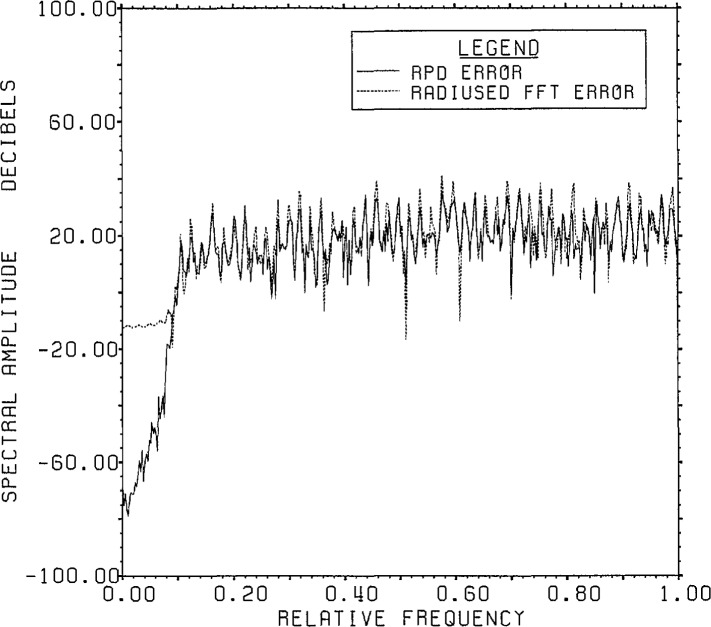
Comparison of errors for root projection and radiused FFT inversion for Example lb. An 800 point FFT was used on the circle of radius 0.997.

Figure 5 shows the comparison of the errors for the estimated inverses from radiused ITT inversion (again padded to 8192 points) and inversion following RPD. In this case radiused FFT inversion produces incorrect answers, not only in the estimate for the inverse, but in the range upon reconvolving the “inverse” with the kernel. The values of the *Y* transform of the noise at the two roots lying inside the radius 0.997 have now greatly perturbed the radiused FFT division result.

The errors for the inverse estimated by RPD are determined by the ratio of the 800 point FFT of the root projected noise to the 800 point FFT of the zero-padded Gaussian. One sees that root projection has preserved the low-frequency features of the signal which are contained in the channels of high signal/noise ratio.

**Figure 6:**
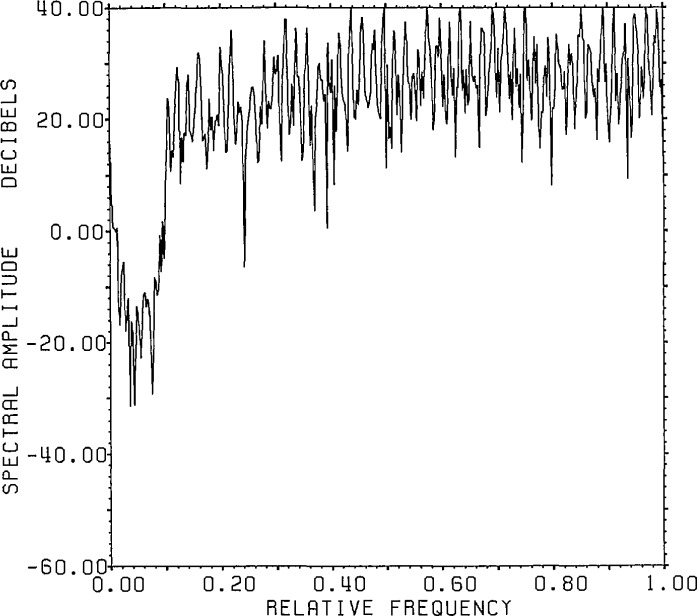
Spectrum from 800 pt FFT (with radius = 1.0) of estimated inverse for projection showing evident break between signal and noise dominated regions.

**Figure 7:**
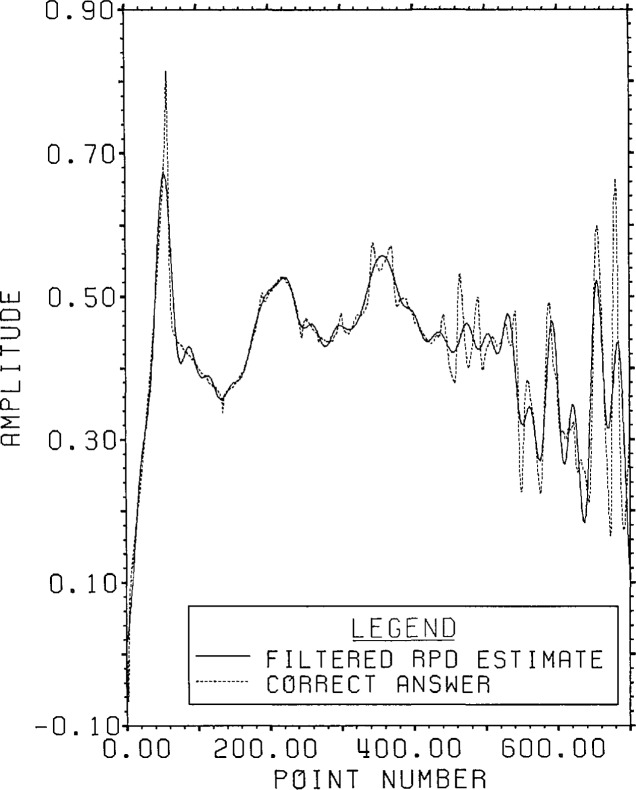
Filtered root projection estimate for Example Ib.

Even without knowing the true noise distribution we can estimate the meaningful channels and build a time domain approximation from them. Figure 6 shows the complete spectrum of the root projected inverse. We can use obvious *a priori* information that all channels beyond 0.08 of the frequency range, where the steep rise begins, are dominated by noise. We use an 801 point optimal filter with a pass band of 0.0 to 0.065 and stopband of 0.08 to 1.0 with attenuation of 90 dB, convolve this with the complete RPD inverse and “trim the ends” to select out the 700 points from point 401 to point 1100. Figure 7 gives the graph of that filtered estimate from RPD. It has an overall r.s.d. of 10.5%, mostly due to errors near the front and back. Throughout much of the first 400 points, the r.s.d. is less than 3%.

A signal-to-noise analysis similar to that for RPD can be carried out for SVD inversion. In this case the channels in which one carries out the analysis are no longer associated with the familiar frequency eigenvectors but are built by the SVD process and are peculiar to the kernel series. However, one has the same number of range dimensions as domain dimensions which makes analysis of the filtering process simpler in SVD than in RPD.

**Figure 8:**
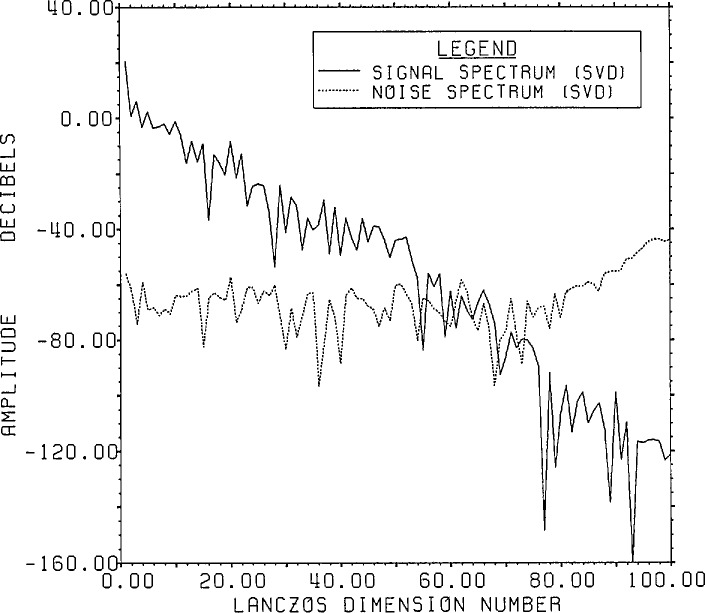
The true signal and noise spectra for Example Ib using the SVD basis (not the Fourier basis) on a Lanczos subspace of dimension 100.

Figure 8 presents the actual signal and noise spectra for the Lanczos/SVD representation in the case of 8 bit range data accuracy. The signal generally falls with decreasing singular values while the noise remains relatively constant at the expected value of −64 dB except for a rise for small singular values (large dimension number). The fraction of the noise which can be represented by only 100 of the 800 range eigenvectors is (799.995/800)2 of the total noise. This highlights one of the features associated with the Lanczos/SVD method, notably the linkage between the range data and the SVD basis whose first element is the signal-plus-noise data vector. Since the SVD basis vectors have a special form, however, it can be difficult to obtain information on the noise statistics for detailed analysis.

**Figure 9:**
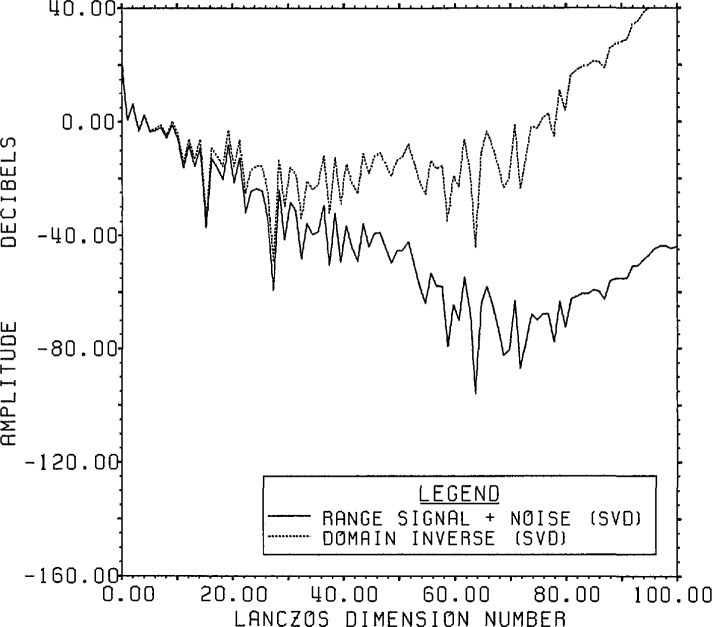
Range signal-plus-noise and estimated inverse spectra for Example Ib using the appropriate SVD bases built in the Lanczos subspace of dimension 100.

**Figure 10:**
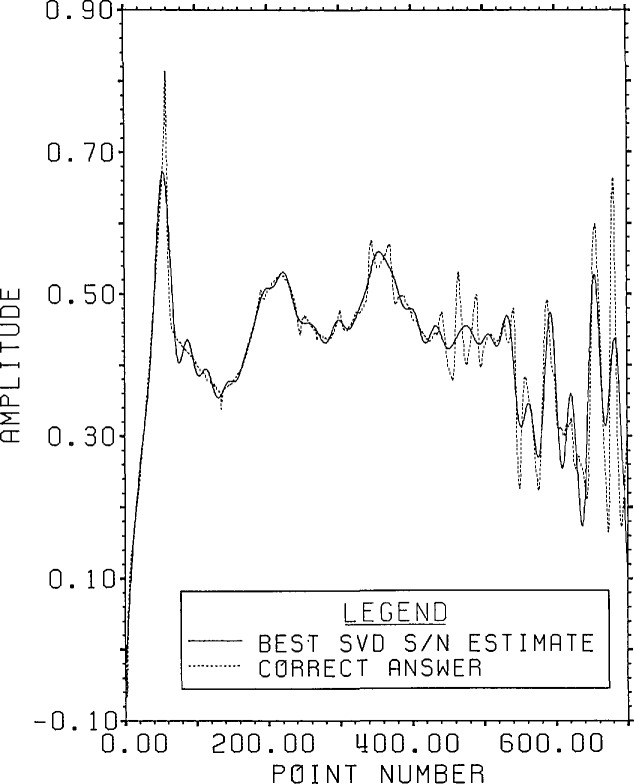
Best Lanczos/SVD estimate for Example Ib using S/N projection.

The plots of signal-plus-noise and inverse SVD spectra are given in figure 9. As can be seen by comparing figures 8 and 9, it is harder to find a best guess from the available data shown in figure 9 than it was for root projection. The technique of truncating above a singular value, which leads to a residual closest to the expected noise residual, is completely in error here. It gives rise to more than 70 terms in the expansion and produces an estimate full of noise with about 1000% r.s.d. from the correct answer. However, based on an expected signal-plus-noise cutoff value of about − 61 dB, a reasonable guess might be to include all components from dimension 1 through 59.^4^ The use of this “reasonable” guess produces the estimate shown in figure 10. The result is remarkably similar to that obtained by filtering of the root projection inverse. It has an overall r.s.d. of 10.4%.

### 3.2 Example II

**Figure 11:**
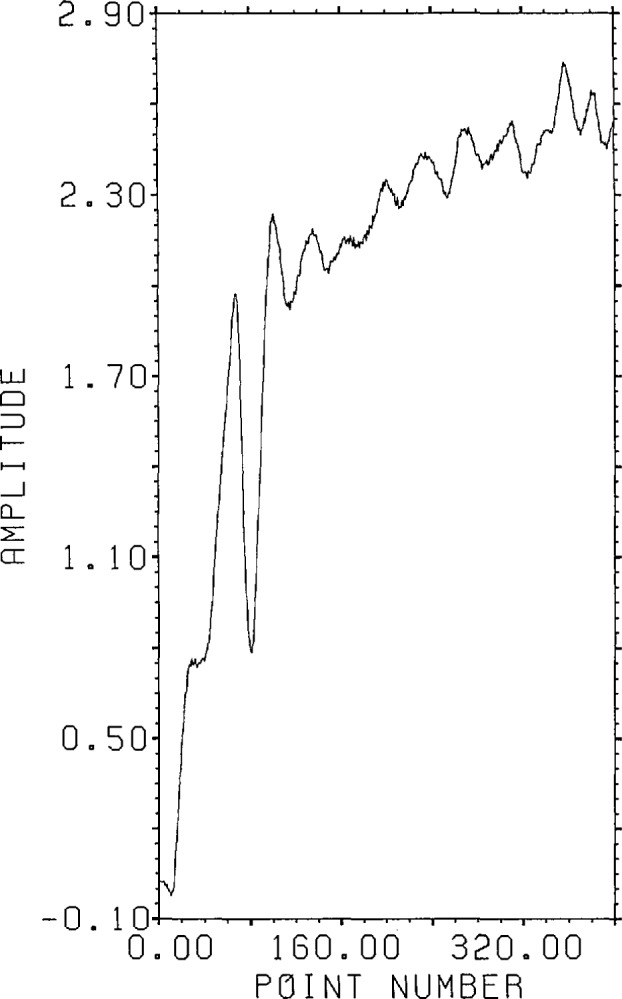
Synthetic output data with noise for model transducer calibration problem (Example II).

**Figure 12:**
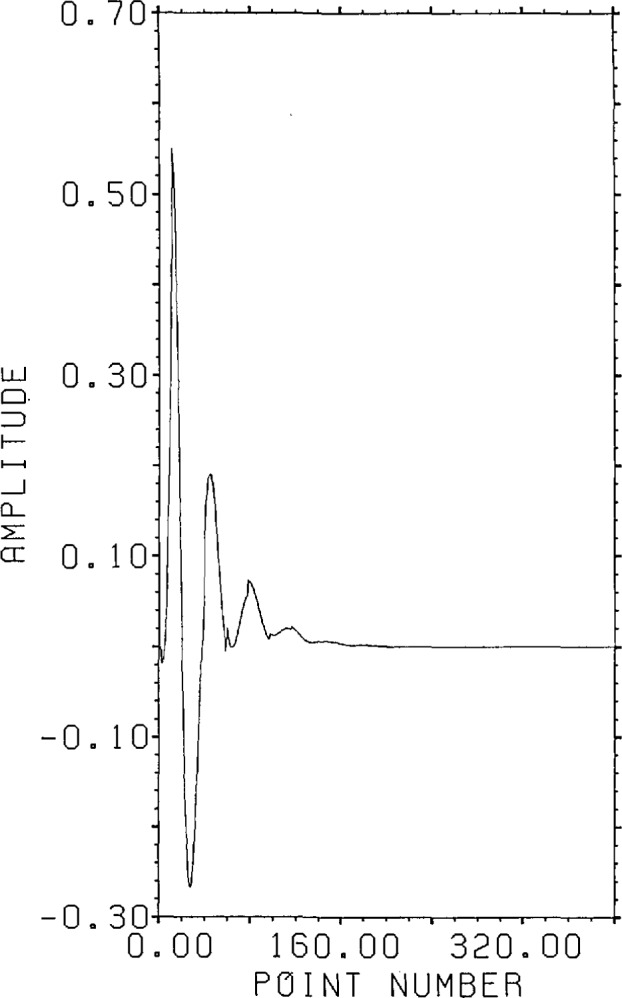
Correct answer for Example II.

The second example concerns deconvolution in the presence of incomplete range information. The kernel for this example is the experimental curve shown in figure 2.4a of reference [1] which was the answer in the previous example [1]. This time only 400 output data points are given as shown in figure 11. The error added was −40 dB random noise, which produces an r.s.d. of about −41 dB. The noise is clearly visible in the figure. The answer, shown in figure 12, represents a possible transducer response function. The output data then represents the response of the transducer to the same stimulus that produced the kernel response of figure 2.4a in reference [1] for the calibrating capacitive transducer.

**Figure 13:**
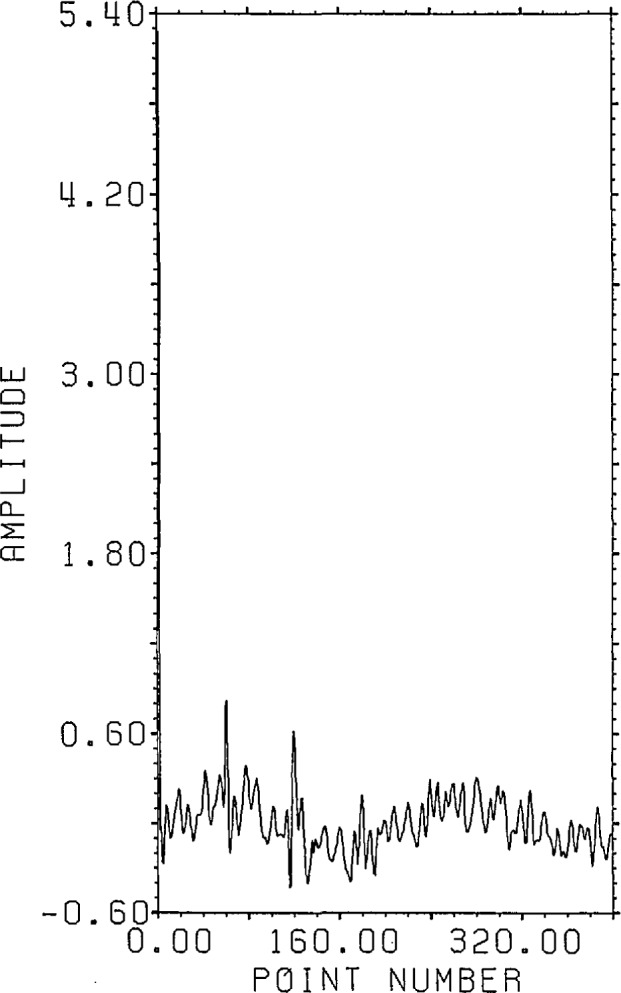
Result of using 800 point FFT division to estimate answer for Example II.

As would be expected in a problem with a rising transient front end, the forward substitution approach is inapplicable, producing relative errors of 5000 on the third point and overflowing soon thereafter. The result of applying FFT division using an 800 point FFT is shown in figure 13. The range r.s.d. for this curve is 158% indicating a completely erroneous result even though the correct answer has less than 200 non-zero points and 400 points were given. If zero padding is added to the FFT (8192 point FFT) the range r.s.d. drops to 44% rather than the 0.8% needed to be within the expected range noise error. The domain r.s.d. against which the SVD and RPD estimates will be compared below is 1976%. Noting that only two roots of the kernel lie below 0.979, one can apply radiused FFT division with a radius of 0.975 to produce a range r.s.d. of 1.1%. The range r.s.d. is almost within acceptable limits, but the domain errors are substantially greater than those produced by either Lanczos/SVD or RPD.

**Figure 14:**
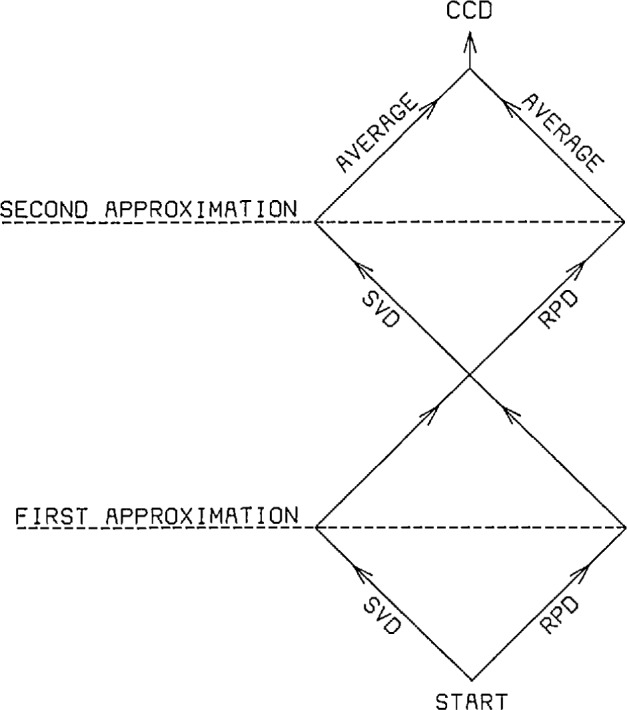
Schematic diagram for cross-cut deconvolution (CCD) algorithm.

To deal with this model problem for which one has only limited *a priori* information (mostly that the answer should not be filled with high frequency noise), we introduce the cross-cutting deconvolution algorithm (CCD) shown schematically in figure 14. This algorithm uses the Lanczos/SVD and RPD methods in tandem to produce a more robust estimate which is at least as good as the particular choice of either estimate and often better than both of these methods. Lanczos/SVD and RPD with optimal filtering are first applied independently to produce first estimates to the deconvolved inverse. Typically, with a kernel whose spectrum is dominated by low frequency components, the SVD estimate will show some of the most prominent high frequency features, but will have reduced low frequency fidelity.

The RPD estimate, on the other hand, will tend to have good low frequency fidelity but reduced resolution of some of the high frequency features and possibly greater end noise. The outputs from each of these algorithms, conservatively filtered to avoid extraneous features, is then fed into the other algorithm as an *a priori* estimate. Each of these two methods uses a different orthogonal decomposition to separate signal from noise and the reuse of the output from one of these algorithms provides essentially no improvement in the estimate (although RPD may be rerun to reduce end noise). However, what one algorithm may discard as noise can contain useful signal when decomposed using the other algorithm. The second, cross-cut estimates are then frequently significant improvements upon the initial estimates. They each, of course, contain induced domain noise which may be partially independent so that the average of the estimates can be expected to provide some overall improvement over the second approximations.

Figure 15a shows the signal-plus-noise and domain spectra and figure 15b the actual signal and noise spectra upon applying Lanczos/SVD inversion to the model problem data. Based on figure 15a with an approximate signal-plus-noise value of −37 dB, reasonable choices might lie between selecting all components from 49 up to 80. As one increases the dimension number past 49, the actual r.s.d. decreases more or less steadily (cf. fig. 15b) from 103% to 65% at dimension number 77. However, as the domain noise increases (and due also to increasing “high frequency” characteristics of the SVD base vectors) more extraneous features become added to the signal. In this case because the eigenvalues are not too small in the 49 to 80 range vis-a-vis those below 49, the “risk” is not too great of introducing a great deal of “high frequency” domain noise by inadvertently adding a channel with excess noise. The only guideline then for choosing among these candidate estimates is *a priori* information about the signal.^5^ We choose the estimate made up from all dimension numbers through 62. This estimate has an overall r.s.d. of 75%. No matter which of these estimates is used as a starting point, the final output of the cross-cutting algorithm varies only between 52% and 54% r.s.d.

The RPD techniques for finding the initial approximation from incomplete range data differ somewhat from those of the SVD method. The potentially large amount of noise that can be introduced by the *a priori* part of the range estimate required by the RPD method can preclude a signal-to-noise analysis similar to that used for SVD. Instead we examine the a posteriori data separately to estimate bounds on the useful part of the frequency range and re-run RPD on windowed raw estimates to remove excessive end noise.

Figure 16a shows the FFT of the 400 points of *a posteriori* data after they have been windowed using a 400 point window built from a maximal ripple filter to smooth the frequency distribution. The figure shows essentially no information above the −40 dB noise level beyond 30% of the Nyquist frequency. Any information about the inverse above the relative frequency of 0.3, then, must either be *a priori* information or information obtained from the SVD estimate. In other cases there will be information over the noise level through high frequency or other “difficult” parts of the frequency range where the kernel has a small spectral amplitude.

With no *a priori* information available we employ a simple extrapolation procedure to create the input range signal for the first RPD approximation. Figure 16b shows the normalized signal-plus-noise and domain spectra after RPD inversion of the given data with the endpoint values continued out to the end of the range data and then windowed. A time weighting of 1:16 (one in the given data and 16 in the extrapolated data) was used to bring the range residuals to well within the expected −41 dB r.s.d. over the initial data. When the weight factor is increased much beyond 20 in this example, exponentially increasing noise terms begin to show up in the inverse and negligible further information seems to be obtainable. In other less noisy or illconditioned cases the weight factor can be increased to several thousand.

**Figure 17:**
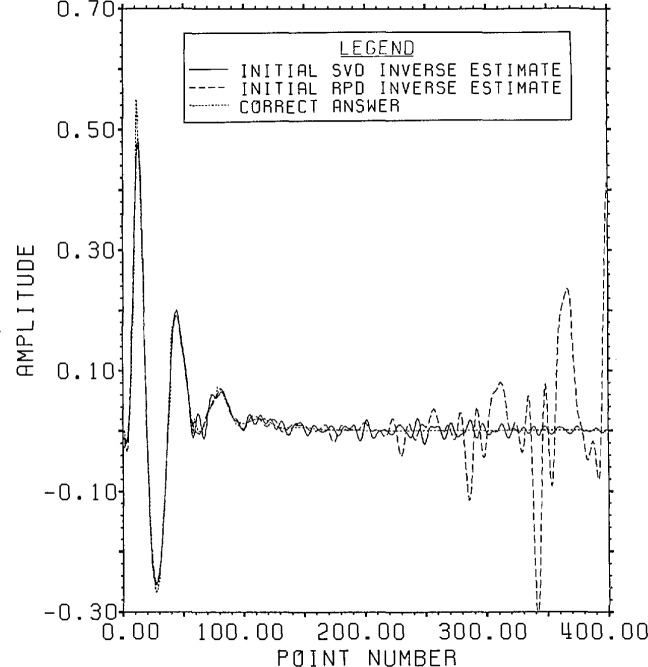
Initial SVD and RPD estimates for Example II.

Figure 16c shows the spectra of the true signal and range noise for the first RPD approximation. The range signal is merely the product of the 800 point FFT’s of the kernel and the answer. The range noise, on the other hand, is not simply the −40 dB *a posteriori* noise, but is here completely dominated by the *a priori* noise which, because of the smooth extrapolation used, has very large low frequency components. Without *a priori* information, there seems to be no simple way of estimating the actual range noise which can be dominated by such errors. The initial procedure for RPD in this case, then, is to choose as broad-band a filter as possible producing an estimate which doesn’t show excessive high frequency noise and to use the general *a priori* information that the inverse should not have a rising high frequency spectrum. No matter which filter is chosen, however, one can expect extensive low frequency errors. How they manifest themselves is shown in figure 17.

Figure 17 shows the SVD and RPD first estimates. As described above the overall r.s.d. for the SVD estimate is 75%. The overall r.s.d. for the RPD estimate is 471%. Examination of the two curves shows this difference to be due to the much greater end error in the RPD estimate. Both of these methods tend to produce larger errors at the end of the time domain since the end values of the answer are involved in fewer terms of the *a posteriori* data. RPD, particularly without a reasonable *a priori* range guess, produces such errors due to leakage of the large *a priori* errors into the *a posteriori* part of the time domain.^6^ It’s easy to confirm in this case that there is almost no valid data beyond point 140 in either estimate. This is done for RPD by using the RPD estimate of figure 17 windowed to 140 points as an *a priori* estimate and reinverting. A lower time weighting ratio of 1:4 is all that’s needed in this case to produce good residuals since the *a priori* range data are more accurate. After filtering the resultant estimate is almost indistinguishable from the original for the first 140 points and has only small oscillations past point 140. This indicates that the large end oscillations in the initial estimate were forced by *a priori* range errors. The overall r.s.d. of the error of this RPD estimate is 60%.

A similar process to show that there is almost no valid data beyond point 140 of the SVD estimate can also be carried out. However, there is nothing intrinsically “wrong” with the estimate produced by the RPD process which returns range residuals almost within the expected noise limits (some increase in residuals is due to filtering). It is only that the *a priori* range data chosen forced features in the answer which were irrelevant to the essential features required by the *a posteriori* data. In fact one can establish a different “model” problem in which the correct answer is the curve given as the RPD estimate in figure 17. Application of SVD to 400 points of range data from this second “model” problem with similar noise added produces estimates similar to those of the model problem as we have presented it with only slightly increased end oscillations. The overall r.s.d. of the error of the similar 62 component estimate from the answer to the alternate problem, for instance, is 366%. The extra data needed to identify the end features of the alternate answer are contained in the last 399 points of missing data.

In most problems with 50% incomplete range data one can still obtain accurate information about the answer through most of the time domain involved. Typical examples we have checked show good accuracy for 60% to 95% of the time range. An assessment of the importance of missing end data may be possible in RPD using knowledge of the positions of the roots in the root transform of the answer vis-a-vis those of the transform of the kernel.

The second stage of the cross-cut algorithm uses each of the two first stage outputs as *a priori* inputs to the alternate algorithm. For this example, since we have established that little meaningful data lie beyond point 140, we will use the windowed first estimates rather than the full estimates as *a priori* inputs. For the SVD process, then, the windowed RPD first estimate is convolved with the kernel and the first 400 points of the resultant time series are subtracted from the *a posteriori* data. Lanczos/SVD deconvolution is then performed on this reduced problem and the estimated inverse added back onto the *a priori* estimate to produce the second estimate.

Figure 18a shows the signal-plus-noise and inverse SVD spectra and figure 18b shows the true signal and noise spectra for this reduced SVD problem. With a −37 dB signal-plus-noise approximate cutoff, a reasonable choice from figure 18a would be a cutoff at 12 vectors, which in this case is confirmed by the curves in figure 18b. The r.s.d. for the second SVD estimate is then 56%.

Figure 19a shows the signal-plus-noise and inverse RPD spectra and figure 19b shows the actual range signal and noise spectra for the reduced RPD problem which has as its *a priori* input the windowed first estimate from the Lanczos/SVD algorithm. The extensive high frequency *a priori* noise level is no longer present, and the apparent cutoff from figures 19a and 19b is between 0.16 and 0.2 relative frequency. We choose the same filter (transition between 0.16 and 0.3 relative frequency) for this stage as in the first stage, since no high frequency noise is apparent. This second RPD estimate has an overall r.s.d. of 60%.

**Figure 20:**
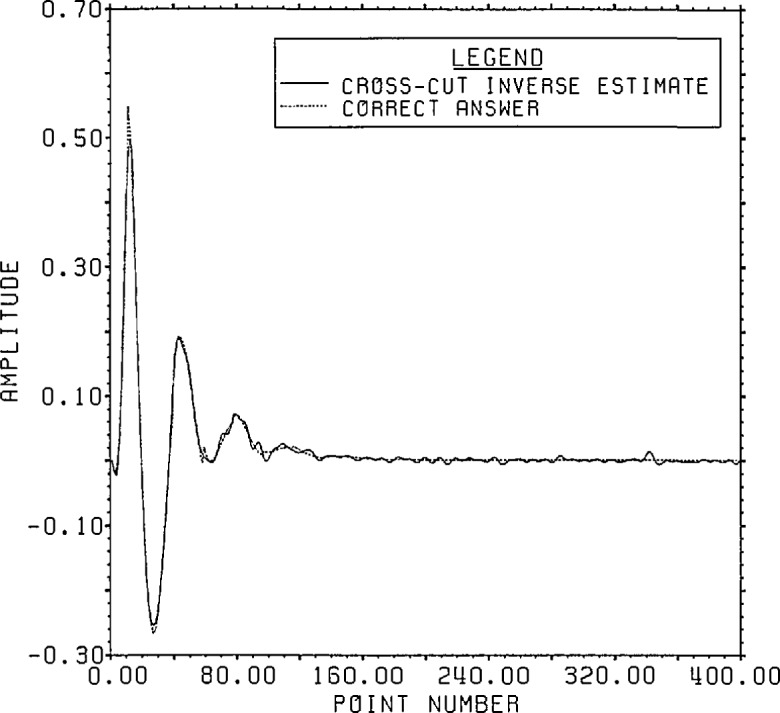
The CCD estimate for Example II.

The final step in the cross-cut algorithm is to average the two estimates arising from the second approximations. The resultant average is shown compared with the correct answer in figure 20. It has an overall r.s.d. of 52%. Since the geometric mean of the answer is 0.014, the actual domain noise is −42.5 dB or 2.5 dB less than the added range noise. Figure 21a gives a comparison of the spectrum of the cross-cut estimate, windowed to 200 points to smooth the spectrum slightly, with that of the answer. Figure 21b gives a similar comparison of the phases. As can be seen from these figures, almost all the error in the estimate is at frequencies above 0.1.

## 4. Summary

A comparative development has been given of a number of related deconvolution methods including a new algorithm based upon root projection. Radiused FFT division, singular valued decomposition (SVD) and root projection deconvolution (RPD) were compared in detail in two examples. The first example involved deconvolution of a 101 point Gaussian filter from a relatively complex 800 point waveform in the presence of noise. Two levels of noise were used. The concept of signal-to-noise filtering was introduced and applied to both SVD and RPD. It was shown to be particularly powerful when used in conjunction with the SVD method. In both cases RPD provided the most accurate inversion estimate although this accuracy was matched by the SVD method for the higher noise level. The second example was a model transducer calibration problem with typically incomplete range data. A robust cross-cut deconvolution (CCD) algorithm utilizing the orthogonal decompositions of both the RPD and SVD methods was used in this example. The deconvolved estimate showed virtually no errors for frequencies below 10% of the Nyquist frequency.

Root transforms were applied in this work only for one dimensional time series. The study of the comparable Riemann surfaces for higher dimensional series or the use of boundary roots in finding signatures for the Radon transforms of such series have yet to be studied.

## Figures and Tables

**Figure 15a:**
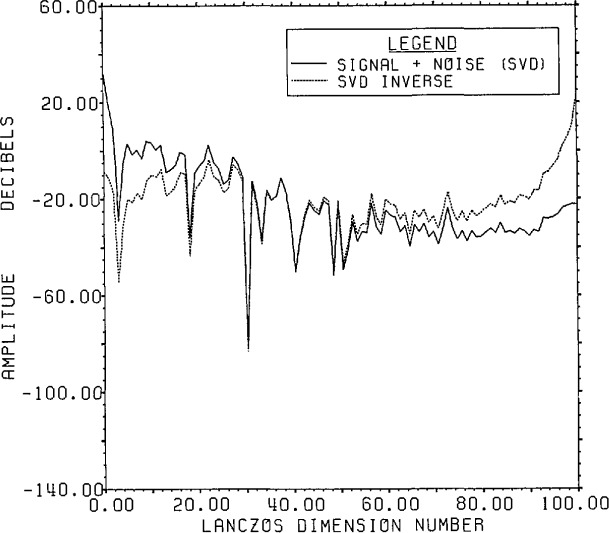
Range signal-plus-noise and estimated inverse spectrum for Example II using the appropriate SVD bases (not the Fourier bases) built in the Lanczos subspace of dimension 100.

**Figure 15b:**
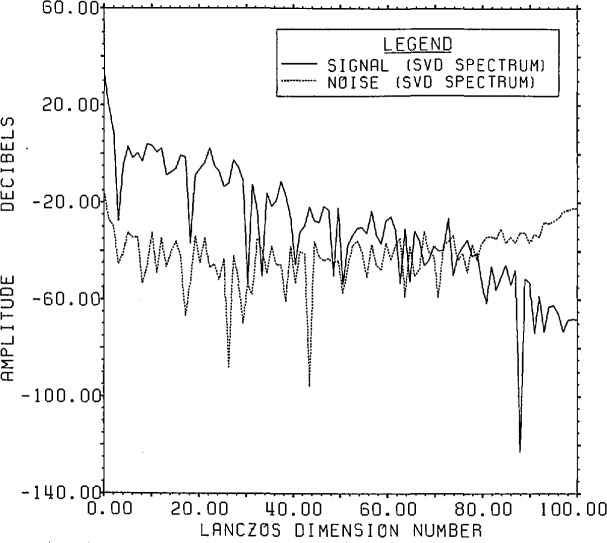
The true signal and noise spectra for Example II using the SVD range basis on the Lanczos subspace of dimension 100.

**Figure 16a:**
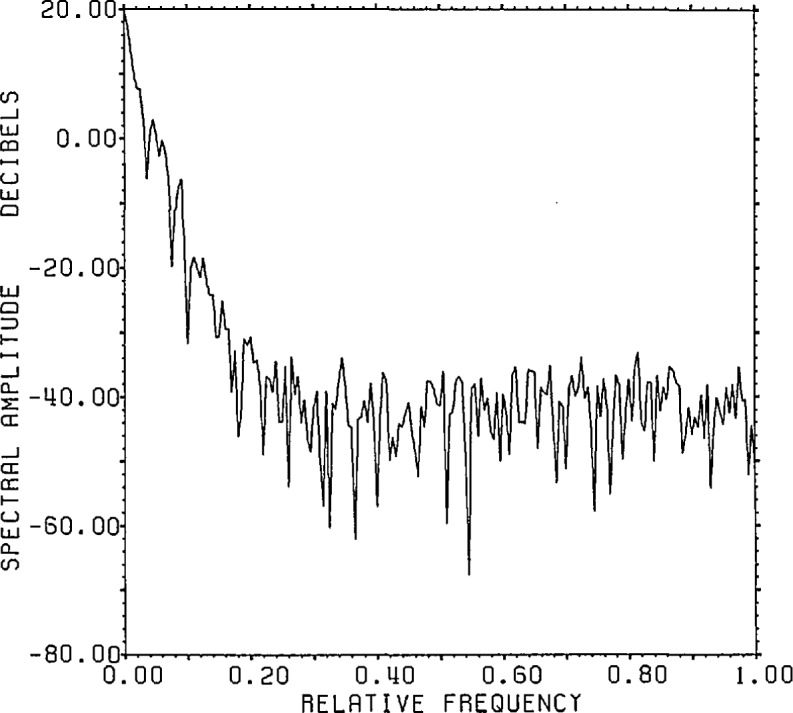
Normalized 400 point FFT of the windowed output data of figure 11.

**Figure 16b:**
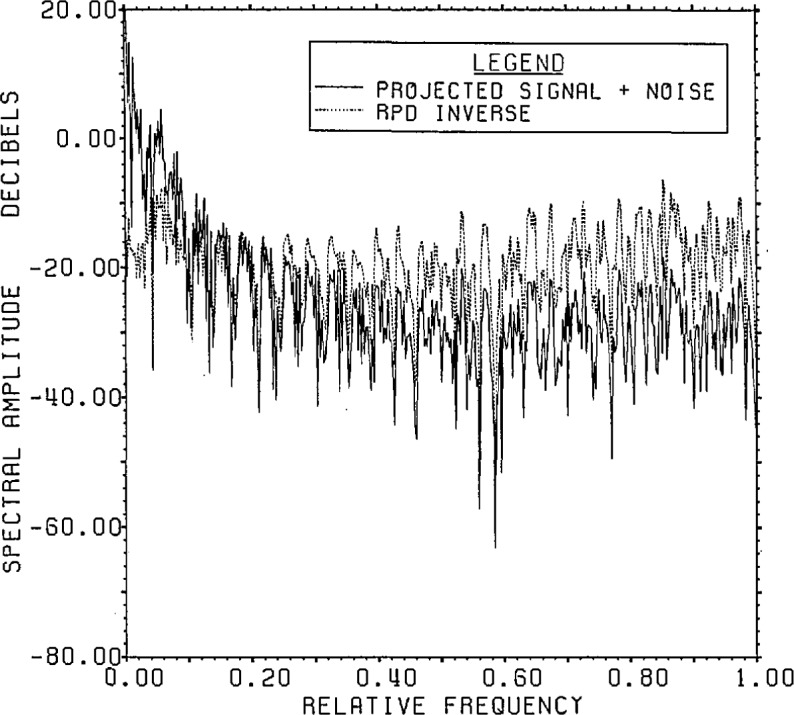
Signal-plus-noise and estimated inverse spectra for example II using an 800 point FFT on the result of root projection where the data of figure 11 were simply end extrapolated out to 800 points.

**Figure 16c:**
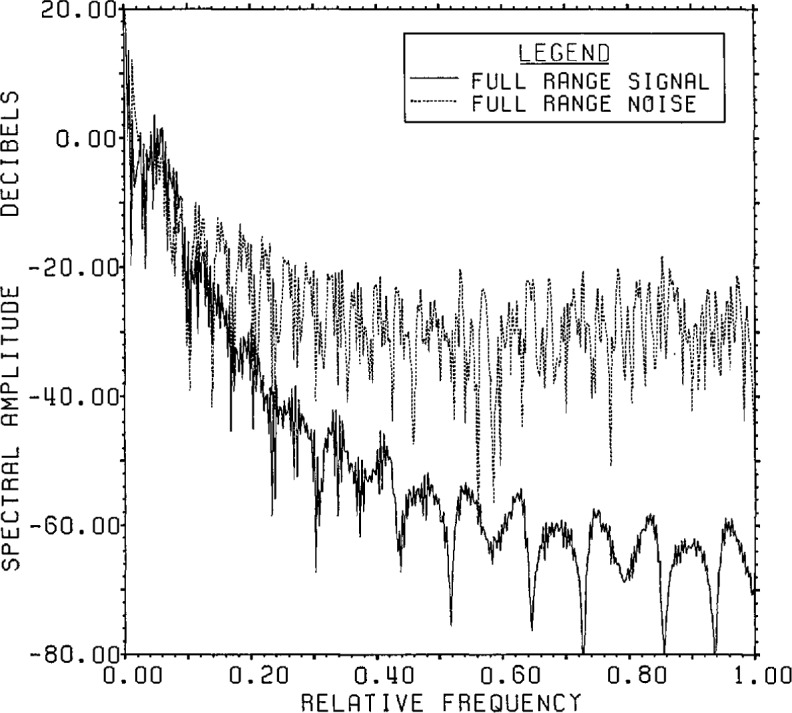
Spectra of 800 point FFT of true range signal for Example II and noise from initial RPD inversion.

**Figure 18a:**
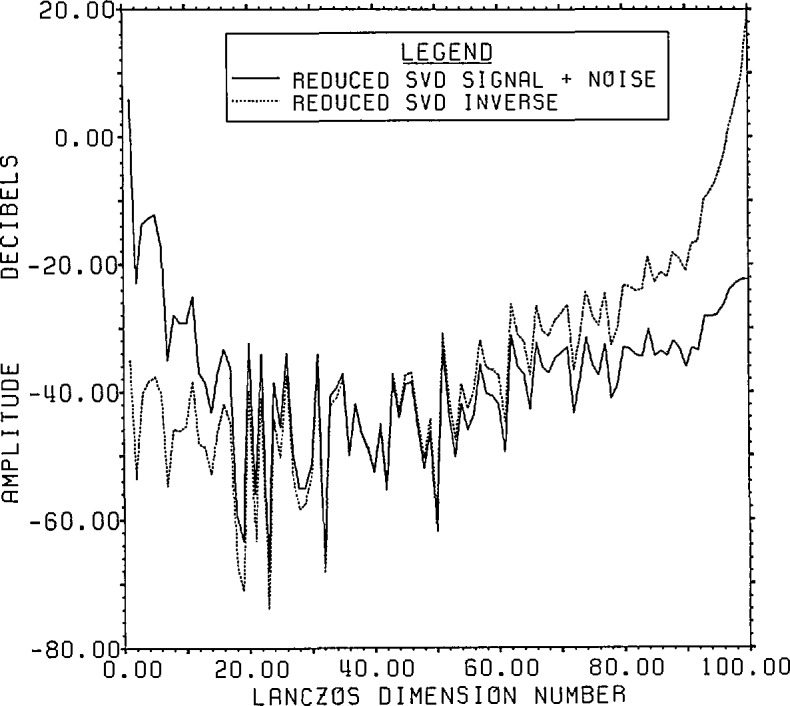
Range signal-plus-noise and estimated inverse spectra for the second stage of the cross-cut algorithm in Example II. The appropriate SVD bases built in the Lanczos subspace of dimension 100 are used. The input is the first stage RPD estimate windowed to 140 points.

**Figure 18b:**
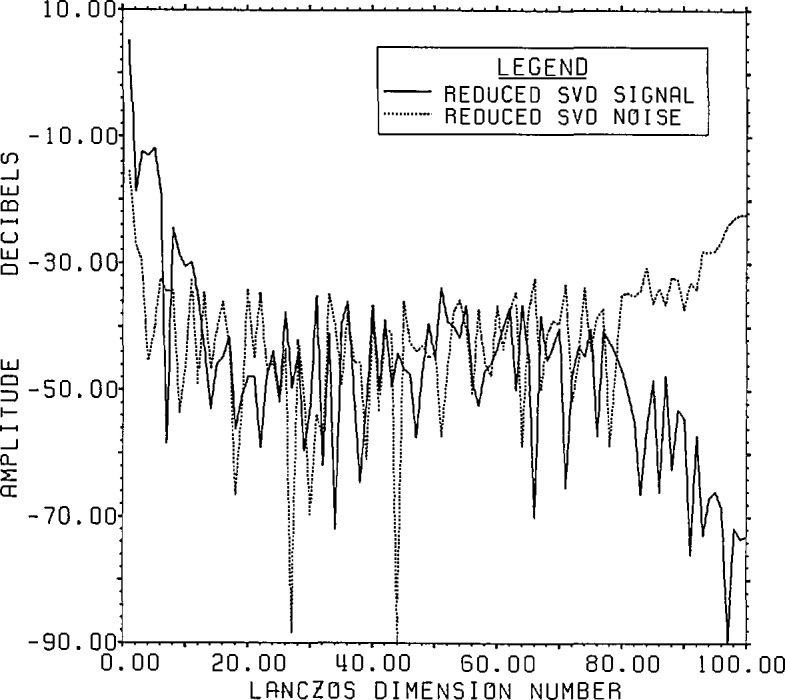
The true signal and noise spectra for the second stage of the cross-cut algorithm in Example II using the SVD range basis on the Lanczos subspace of dimension 100.

**Figure 19a:**
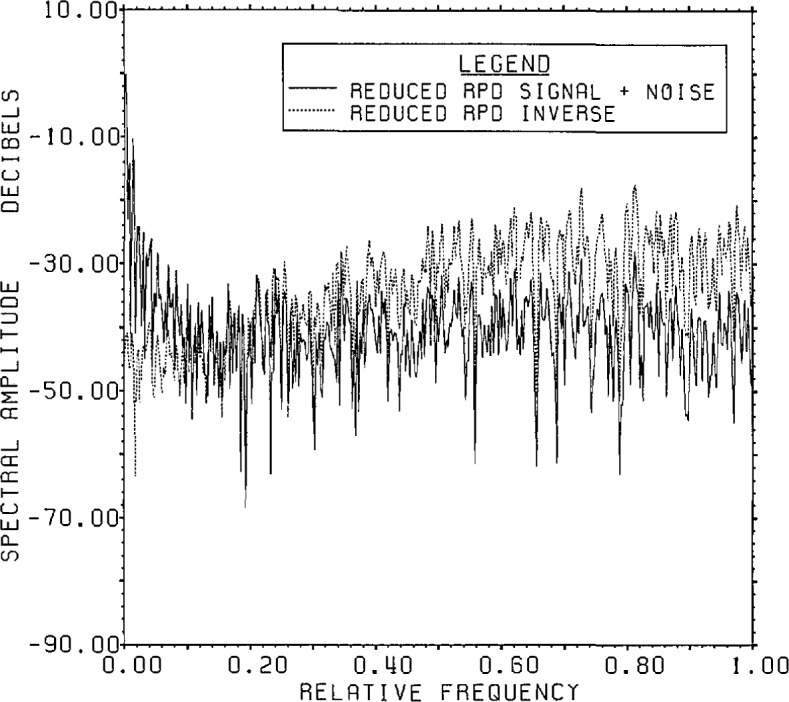
Signal-plus-noise and estimated inverse spectra for the second stage of the cross-cut algorithm in Example II. An 800 point FFT is used on the result of root projection from the windowed first stage SVD input.

**Figure 19b:**
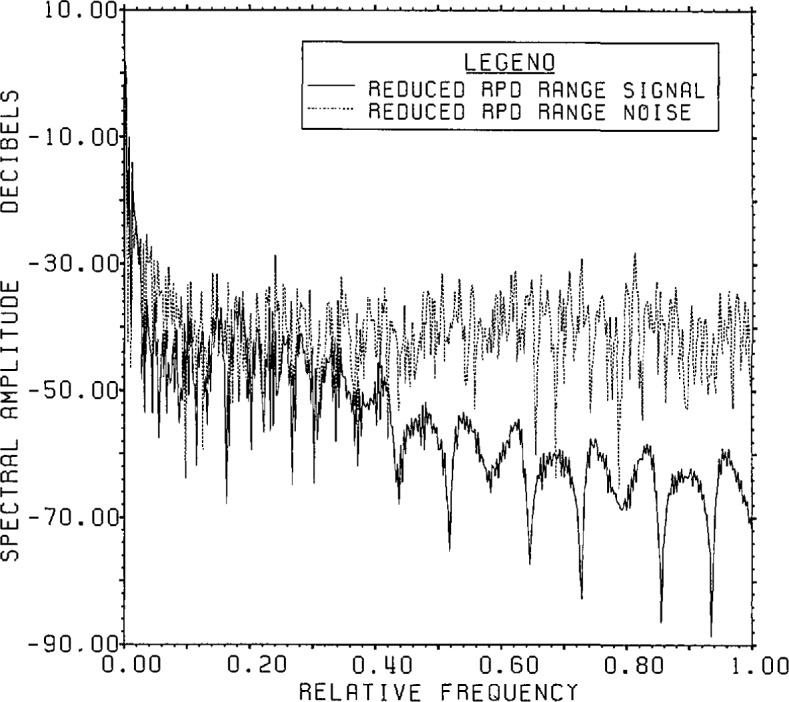
The true range signal and noise spectra for the second stage of the cross-cut algorithm in Example II. An 800 point FFT is used on the result of root projection from the first stage SVD input.

**Figure 21a:**
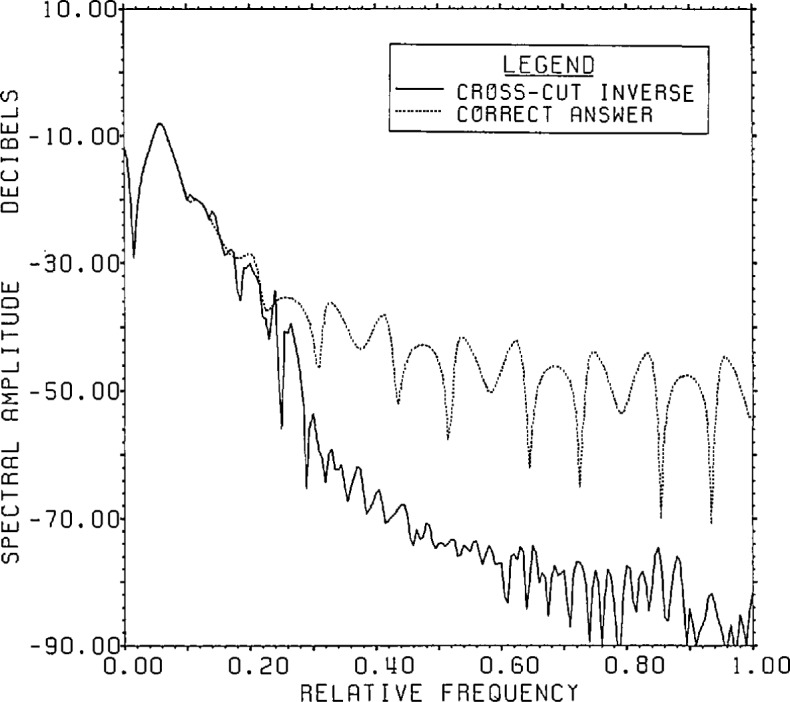
Spectrum of the CCD estimate for Example II.

**Figure 21b:**
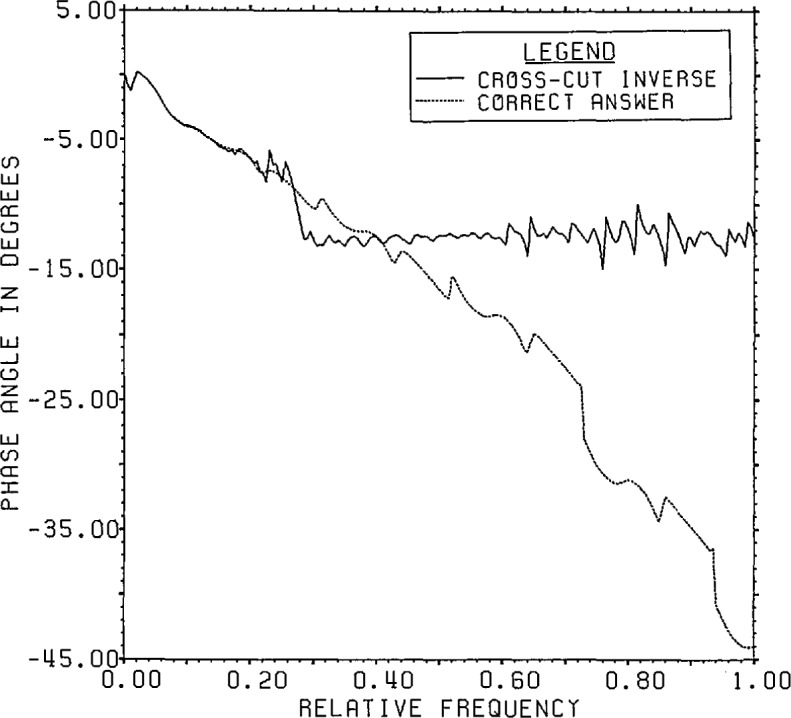
Phase of the CCD estimate for Example II.

## Footnotes

1 We use here the modified Dirac bracket and *Y* transform notations employed in references [1] and [2].

2 In the ideal continuous case there is no truncation or aliasing problem associated with *υ*(*y*), only the noise problem. In that case the Fourier transform for *β*(*y*) should be entire in the upper half plane, and the exponential noise buildup and non-causal representation arguments against the continuous Stokes’ deconvolution formula for dividing the appropriate Fourier transforms are still valid [3].

3 The lack of independence of the FFT components in the image space means that projecting out some components will produce inverses no longer zero beyond point #700, thus no longer satisfying the multiplication of FFT’s rule of the space of dimension *M.* Signal-to-noise analysis, then, is not rigorously correct in RPD on a channel by channel basis. To avoid this complication, filtering for RPD will be carried out in the domain space after the inversion. This change of dimension before filtering implies *a priori* information about the carry-over of “relevant” frequency features between FFT representations of different cardinality.

4 One could also use a S/N cutoff at −61 dB; the resultant estimate in this case is visually identical to that chosen. However, we have found by experience that something like a Gibbs phenomena also occurs in SVD expansions so that including all components up to a given dimension number tends to give slightly less oscillations in the estimates than using a S/N cutoff. A regularization type approach to this problem would be to use all channels for the inversion, but to replace each singular value, λ, by λ + ϵ where e is some appropriately chosen small constant.

5 The Lanczos dimension used throughout this paper is 100. However, a special run was made for this example using Lanczos dimension 200. Although the error in representation of the answer was much better in the 200 case, the range noise was sufficiently large that only small improvement occurred in the r.s.d. of the estimates. The main improvement was in the signal-plus-noise diagram which showed a distinct plateau now making the choice of dimension number 77 as a cutoff almost apparent.

6 Errors also tend to occur very near the causal time boundary in both methods when extensive projection is employed. One can give a heuristic interpretation of this front end error for RPD based on the fact that the RPD estimate is low-pass filtered. By reducing the frequency range by a factor of *M*, say, lower resolution occurs in the time domain which shows up most clearly in the first *M* points of the time domain.
